# Totally endoscopic inguinal hernia repair: A new approach

**DOI:** 10.1016/j.ijscr.2019.08.008

**Published:** 2019-08-19

**Authors:** Pablo Medina, Facundo Iriarte, Guido Luis Busnelli, Facundo Orrego, Daniel Enrique Pirchi

**Affiliations:** a Abdominal Wall Surgery Department, Hospital Británico de Buenos Aires, Perdriel 74, CABA, 1280, Argentina; b Program Director of General Surgery Department, Hospital Británico de Buenos Aires, Perdriel 74, CABA, 1280, Argentina

**Keywords:** Endoscopic, Minimally-invasive, Lichtenste, Inincisional hernia

## Abstract

**Background:**

Minimally invasive approach is used to repair as many defects of the abdominal wall as possible with the same approach, with the advantages implied by this way to perform surgery. Nowadays, efforts are made to create a minimally invasive approach to cope with the necessity to repair different defects of the abdominal wall at the same time.

**Methods:**

A minimally invasive approach, with long standing endoscopic and laparoscopic principles on a well stablished inguinal hernia repair technique (Lichtenstein), is used to create a new approach to restore a unilateral inguinal hernia with the possibility to perform an adjunctive approach to solve other defects of the abdominal wall, in this case midline incisional hernia. A written consent was previously obtained, and Institutional Review Board approval was not needed.

**Results:**

We performed an endoscopic repair of a right inguinal hernia, and a laparoscopic approach using the same surgical site to repair a midline incisional hernia. Patient was discharged at 36 hs post-procedure. Sixth month later the patient does not have evidence of a recurrence of either defect.

**Conclusion:**

This new approach has proved to be feasible, giving the advantages of a minimally invasive approach, but more experience and time are needed before getting objective and real conclusions.

## Highlights

• New minimally invasive approach for combined defects of the abdominal wall.
• Endoscopic approach for one side inguinal hernia and midline abdominal wall defect.
• First successful experience with no recurrence in first six months.
• Same advantages of minimally invasive approach in the new technique.
• More experience is required in a high laparoscopic volume center.

## 1 Introduction

Since its description in 1984, Lichtenstein inguinal hernia repair has become the gold standard technique, being a pure prosthetic, tension free repair, which achieved consistently low recurrence rates in long term outcomes analysis [1]. After that when the laparoscopic approach began to show its advantages, it started to be used in the resolution of inguinal hernias, especially in bilateral ones [2].

In this era where the minimally invasive approach is intended to be used in several surgical techniques, a new approach is created where the minimal number of access are used to repair a unilateral inguinal hernia associated with a midline defect of the abdominal wall, where the space between the mayor and minor oblique muscle is used to access the inguinal canal, and the same ports used to enter the abdominal cavity and solve an incisional hernia of the midline abdominal wall. This case report has been reported in line with the SCARE checklist [3].

## 2 Presentacion of case

**Fig. 1:**
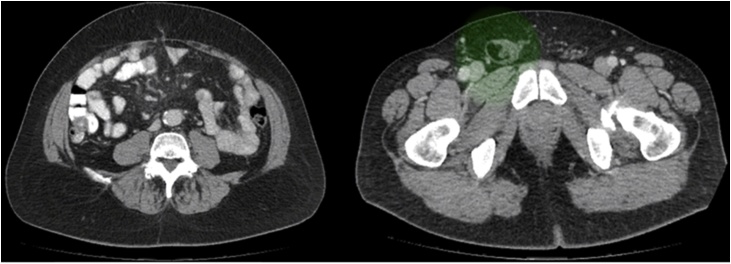
CT Abdomen: right inguinal hernia and umbilical hernia.

A 65-year-old man with history of a conventional appendectomy, TAPP laparoscopic left inguinal hernia repair and hypertension was referred to our department for a right inguinal hernia associated with abdominal discomfort. Physical exam showed an umbilical incisional hernia without complications and a bulging of the midline above the umbilicus that caused the patient an esthetic discomfort. CT confirmed the diagnosis (Fig. 1).

We decided to perform an endoscopic technique repair of the right inguinal hernia, approaching the inguinal canal dissecting the space between major and minor oblique muscles.

**Fig. 2:**
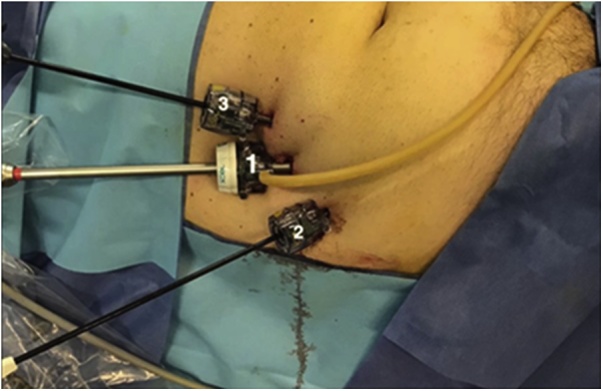
Position and order of placement of trocars.

**Fig. 3:**
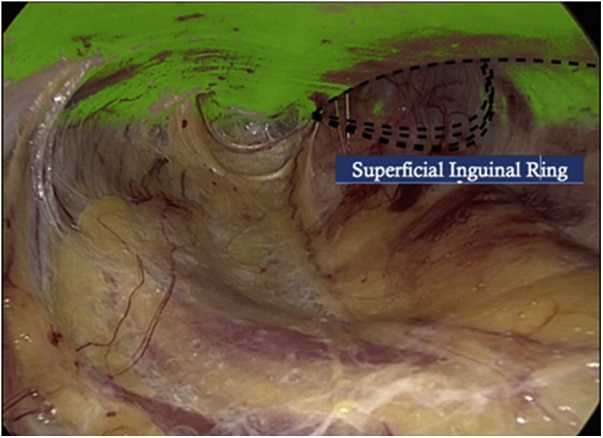
External oblique muscle and superficial inguinal ring.

**Fig. 4:**
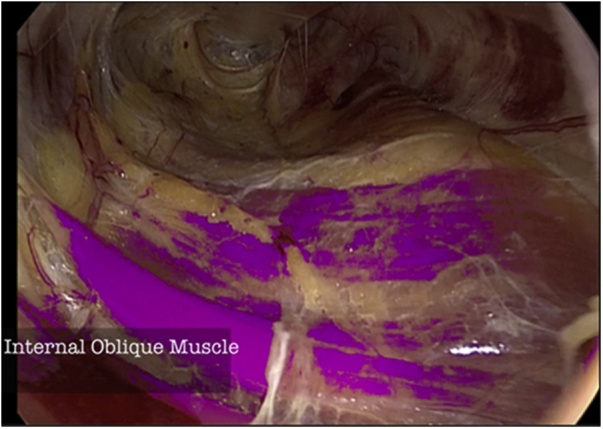
Internal oblique muscle.

Under general anesthesia, the patient is positioned in supine position and the surgeon is located next to the patient on the right side. The monitor is located behind the patients feet and the assistant next to the surgeon. A 10 mm incision is made at the right flank, rome dissection to reach major oblique muscle, incision and dissection until we see minor oblique muscle. At this point we introduce the opti view trocar and, with the help of co2, a rome dissection between this two muscles in caudal direction is made until we create a cavity with enough space to place a 5 mm trocar to introduce a grasper first and a second one later achieving triangulation (Fig. 2), and with rome maneuvers we approach the inguinal canal. A space is created where the surgeon can see, on the top of the screen the External Oblique muscle and the Superficial Inguinal Ring (Fig. 3), and on the bottom of the screen, the Internal Oblique muscle (Fig. 4).

**Fig. 5:**
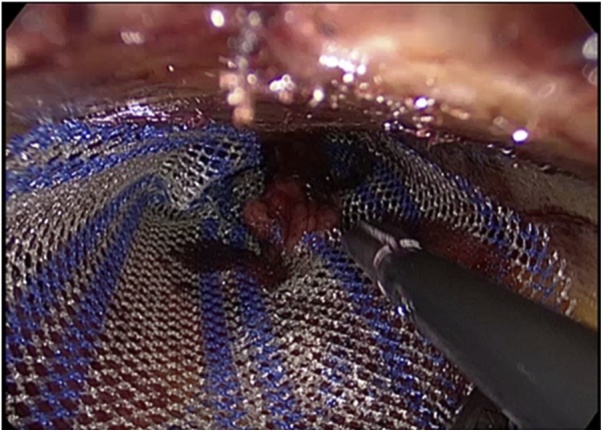
Mesh placement.

The inguinal cord is individualized and dissected free. Then, an incision is made in the cremaster muscle in order to localize the sac and a lipoma next to it, the lipoma is removed and an endoloop of absorbable suture is placed at the base of the sac, and this is sectioned and removed too. The space surrounding the cord is dissected so as to visualize the pubic tubercle and to create an appropriate space to introduce and place the mesh prosthesis of 13 × 8 cm as in Lichtenstein repair (Fig. 5). The fixation of this one is achieved by absorbable tacks (Securestrap- Ethicon) first to the pubic tubercle, and then to the superior aspect of the mesh.

Once the inguinal hernia repair is finished, umbilical incisional hernia laparoscopic repair follows. The monitor is placed on the left side of the patient, pneumoperitoneum is achieved by placement of Veress needle through Palmer’s point. We take advantage of the incisions made and use the 10 mm incision to enter the abdominal cavity with opti view trocar. An additional 5 mm incision is made at the right upper quadrant (RUQ) to insert a trocar and achieve a correct triangulation. The umbilical defect is seen without content apart from fat from the round ligament. Parietal peritoneum is dissected free and an intraperitoneal mesh (Proceed 15 × 15 cm) is placed to cover the defect, firstly attached with two cardinal trans parietal points of non-absorbable suture to the abdominal wall, then with double layer of non-absorbable tackers (Pro Tack Fixation Device). Finally, the 10 mm incision is closed. No drainage is placed in cavity, nor in the space between the oblique muscles.

The patient underwent an uneventful recovery and was discharged at 36 hs post-procedure. During the first month of follow up the patient had no complications and coursed it without pain. At a later control at the sixth month the patient does not have evidence of a recurrence neither of the inguinal hernia nor the incisional one. The patient has full range of his normal activities.

## 3 Discussion

We sought to combine a minimally invasive approach with long-standing endoscopic and laparoscopic principles [2] on a well stablished hernia repair technique thus avoiding the opening of the peritoneum to achieve the mesh placement for the repair of the inguinal hernia, and therefore seeking to avoid the possibility of certain complications. We took advantage of the trocars site to solve the problem of an additional pathology laparoscopically such as an umbilical incisional hernia.

Since its description in 1984, Lichtenstein inguinal hernia repair was the first pure prosthetic, tension free repair to achieve consistently low recurrence rates in long term outcomes analysis. From that on, this technique was the procedure of choice to repair inguinal hernias in a conventional way. Some of the advantages of this technique are the use of regional anesthesia, less operative time, and reduced costs.

Later, laparoscopic approach would bring several advantages to the inguinal hernia repair, including the possibility to simultaneously repair a bilateral hernia, the reduction of pain in the postoperative period, and the earlier return to daily activities. All of this advantages are given by a minimally invasive approach to surgery [4,5]. Nowadays, both Lichtenstein repair and laparoscopic, TAPP or TEP, are the gold standard for inguinal hernia repair, and the choice of either depend on the expertise of the surgeon and the resources available at the care center.

When performing this endoscopic inguinal hernia repair, the first thing the surgeon notice is the perfect view of the anatomy of the region, which brings the opportunity to observe the nerves and spare them in the dissection. Also, there is scant hemorrhage in the dissection if any, which allows the perfect view of the region. The space created is enough to place a mesh of the same size as those used in mentioned techniques. If performed alone it could be used with regional anesthesia.

The lack of consensus as to the optimum repair technique, demonstrates that the perfect technique is yet to be discovered and efforts are made to combine the best aspects of the existing ones to perform a technique that covers the necessity of the patients, providing them a long term, durable result with the advantages of a minimally invasive technique, returning them to active life as soon as possible.

## 4 Conclusion

The aim of this novel technique is to seek the advantages of a well-known Lichtenstein inguinal hernia repair from a minimally invasive point of view, and take advantage at the same time of the surgical site to combine it with a laparoscopic approach to solve an additional pathology, in this case an umbilical incisional hernia. In the short period of follow up of one month and the sixth month there were no complications. More experience and time are needed before getting objective and real conclusions.

## Funding

There were no sources of funding.

## Ethical approval

Ethical approval has been exempted by our institution.

## Consent

Written informed consent was obtained from the patient for publication of this case report and its accompanying images.

## Guarantor

Pablo Medina accepts full responsibility for the article.

## Provenance and peer review

Not commissioned, externally peer-reviewed.

## CRediT authorship contribution statement

**Pablo Medina:** Conceptualization, Methodology, Investigation, Supervision, Writing - original draft. **Facundo Iriarte:** Investigation, Writing - original draft, Writing - review & editing, Visualization. **Guido Luis Busnelli:** Visualization, Resources. **Facundo Orrego:** Visualization, Resources. **Daniel Enrique Pirchi:** Validation, Supervision, Project administration.

## Declaration of Competing Interest

Pablo Medina, Facundo Iriarte, Guido L. Busnelli, Facundo Orrego, and Daniel E. Pirchi declare that there is no conflict of interest regarding the publication of this article.
